# Appendectomy due to lead poisoning: a case-report

**DOI:** 10.1186/1745-6673-3-23

**Published:** 2008-10-17

**Authors:** S Mohammadi, AH Mehrparvar, M Aghilinejad

**Affiliations:** 1 Department of Occupational Medicine and Occupational Medicine Research Center of Iran University of Medical Sciences, Shaheed Hemmat highway, Tehran, Iran; 2 Department of Occupational Medicine, Yazd University of Medical Sciences, Yazd, Iran

## Abstract

**Background:**

Lead poisoning is a common occupational health hazard in developing countries and many misdiagnoses and malpractices may occur due to unawareness of lead poisoning symptoms.

**Case presentation:**

We report a case of occupational lead poisoning in an adult battery worker with abdominal colic who initially underwent appendectomy with removal of normal appendix. Later on he was diagnosed with lead poisoning and was treated appropriately with lead chelator (CaNa_2_EDTA).

**Conclusion:**

Lead poisoning is frequently overlooked as the differential diagnosis of acute abdomen which may result in unnecessary surgery. Appropriate occupational history taking is helpful in making a correct diagnosis. Occupational lead poisoning is a preventable disorder and a serious challenge for the health and labor authorities in developing countries.

## Background

Lead is present in trace amounts in all soils, water, and foods. Currently, lead is used in more than 900 industries, including mining, smelting, refining, battery manufacturing, soldering, and so on. [1]

Lead toxicity today is recognized as a major environmental health risk, with the most serious effects in young children. [1] But owing to insufficient controlling measures in work places, lead poisoning is yet a common occupational health hazard in developing countries and many misdiagnoses and malpractices can occur due to unawareness of lead poisoning as an imitator of many organ symptoms. [2]

## Case-report

Our patient is a 41 year-old married male (with 3 children, the eldest being 7) living in Tehran. His medical history did not show any other disease or hospitalization. He is a heavy smoker (about 30 pack-year). He has been working as an operator of a machine used to cut and finish lead plates for 14 years in a battery-manufacturing plant. He used to work in a lead smelting plant for 2 years before his current job.

He has had severe abdominal colic since 4 months ago. He was admitted in a hospital with the diagnosis of appendicitis and underwent an appendectomy operation (pathology revealed normal tissue of appendix) without any improvement in symptoms. He has also had other symptoms including headache, lethargy, fatigue, irritability, insomnia, muscle pain (especially in the legs), constipation, decreased libido, nausea, vomiting, tremor, loss of appetite, and weight loss.

After discharge from hospital without any improvement, he was referred to occupational medicine clinic of Tehran University of Medical Sciences with suspicion of lead intoxication by an occupational medicine specialist who was in charge of medical examinations of the workers in that plant.

When we visited him, he had the aforementioned abdominal pain. Upon physical examination he was afebrile (37.2°C oral) with respiratory rate of 14/min and pulse rate of 82/min. His blood pressure was 145/90 mmHg. His conjunctivae were pale; he had a mild tenderness in deep abdominal palpation and a surgery scar on his right lower quadrant. Blood test revealed a blood lead level of 118 μg/dl. However, he didn't have any other symptoms such as lead lines or symptoms related to neuropathy. The results of other laboratory tests are as follows:

Three months after appearance of symptoms: WBC 6.8 × 10^3^, RBC 4.3 × 10^6^, Hb 10.9, Hct 35.1, MCV 80.1, MCH 24.9, MCHC 31.1, PLT 255 × 10^3^.

One month later (after admission): Hb 9.7, Hct 29.8, MCV 81, MCH 26.4, MCHC 32.5.

He was treated with continuous IV infusion of CaNa_2_-EDTA 1 g Bid for 5 days. During treatment his renal function was evaluated on a daily basis. After starting the treatment his symptoms improved and he was discharged from hospital. After 2 weeks his blood lead level was 38.3 μg/dl. Upon complete recovery he returned to his job at his former workplace.

## Discussion

Lead intoxication is highly prevalent among persons chronically over-exposed to lead. Symptoms include arthralgia, myalgia, headache, weakness, depression, loss of libido, impotence, and vague gastrointestinal problems. [3] The first gastrointestinal symptoms begin to appear at blood lead level around 80 μg/dl. They consist of loss of appetite, digestive disturbances, epigastric discomfort after meals, and either constipation or diarrhea. When the blood lead level exceeds 100 μg/dl, the likelihood of more severe symptoms increases. These include occasional or frequent abdominal colic and severe constipation. If exposure does not stop, classic lead colic develops [4], which often results in inappropriate laparatomy. [5]

In our country, the main sources of occupational lead poisoning are battery-manufacturing plants, lead smelting plants, and steel plants. We do not have any specific limit values and use ACGIH-TLVs as regulatory measures for blood lead (50 μg/dl in two occasions needs removal from work).

Our patient worked in a battery-manufacturing plant and had typical symptoms and signs of lead poisoning; he was also inappropriately operated for appendicitis.

Lead intoxication symptoms such as abdominal pain, constipation, nausea, vomiting, etc make this disease an important diagnosis to be differentiated from many gastrointestinal and surgical diseases, and the significant point is that lead intoxication is preventable and its treatment is straightforward. Therefore, paying attention to a good occupational history will prevent many unnecessary and/or avoidable medical interventions.

## Competing interests

The authors declare that they have no competing interests.

## Authors' contributions

SM and AHM contributed in visiting the case, MA contributed in editing the manuscript, all authors contributed in drafting the manuscript, all authors read and approved the final manuscript.

## Consent

Written informed consent was obtained from the patient for publication of this case report. A copy of the written consent (in Persian) is available for review by the Editor-in-Chief of this journal.
